# Antihypertensive Therapy and Incidence of Cancer

**DOI:** 10.3390/jcm11226624

**Published:** 2022-11-08

**Authors:** Sven H. Loosen, David Schöler, Mark Luedde, Johannes Eschrich, Tom Luedde, Niklas Gremke, Matthias Kalder, Karel Kostev, Christoph Roderburg

**Affiliations:** 1 Clinic for Gastroenterology, Hepatology and Infectious Diseases, University Hospital Düsseldorf, Medical Faculty of Heinrich Heine University Düsseldorf, 40225 Düsseldorf, Germany; 2 KGP Bremerhaven, 27574 Bremerhaven, Germany; 3 Department of Hepatology and Gastroenterology, Charité University Medicine Berlin, Augustenburger Platz 1, 13353 Berlin, Germany; 4 Department of Gynecology and Obstetrics, Philipps-University, 35043 Marburg, Germany; 5 Epidemiology, IQVIA, 60549 Frankfurt, Germany

**Keywords:** antihypertensive therapy, cancer, diuretics

## Abstract

**Background:** Antihypertensive pharmacological therapy includes diuretics, beta-blockers, ACE inhibitors, calcium channel blockers and angiotensin II receptor blockers. Besides their use in arterial hypertension, these drugs also play a major role in the therapy of portal hypertension, heart failure and coronary artery disease. Systematic analyses on the possible influence of these medications on cancer incidence are lacking. **Methods:** By utilizing the Disease Analyzer database (IQVIA), 349,210 patients with antihypertensive drug prescriptions between 2010 and 2020 without a diagnosis of cancer prior to or at the date of initial drug prescription were included. Propensity score matching was carried out by 1:1:1:1:1 according to the five antihypertensive treatments. Cox regression analyses were performed to investigate an association between antihypertensive drugs and the incidence of cancer. **Results:** Patients who were diagnosed with cancer were treated with diuretics in 19.9% of cases, calcium channel blockers in 16.9% of cases, and angiotensin II receptor blockers, ACE inhibitors, or beta-blockers in 13.9%, 13.2% and 12.8% of cases, respectively. Cox regression models revealed that diuretic use positively correlated with liver cancer incidence (HR: 1.31, 95%CI: 1.12–2.63) and lymphoid/haematopoietic tissue cancer incidence (HR: 1.27, 95%CI: 1.10–1.46). Use of diuretics negatively correlated with the incidence of prostate (HR: 0.64, 95%CI: 0.53–0.78) and skin cancer (HR: 0.81, 95%CI: 0.72–0.92). Finally, a positive association was found between angiotensin II receptor inhibitors and prostate cancer incidence (HR: 1.50, 95%CI: 1.28–1.65). **Conclusions:** These data suggest that diuretic use might be associated with liver cancer and lymphoid/haematopoetic tissue cancer development.

## 1. Introduction

Antihypertensive drugs, including diuretics (DIU), beta-blockers (BB), ACE inhibitors (ACEI), calcium channel blockers (CCB) and angiotensin II receptor blockers (ARB), are commonly prescribed worldwide. Between 1990 and 2019, the number of people aged 30–79 years with hypertension has increased from 648 million to 1.278 billion [1], while incidence rates between 3 and 18% have been reported [2]. In addition to arterial hypertension, these drugs are recommended for the treatment of heart failure, coronary artery disease, liver cirrhosis with ascites or essential tremor, just to name a few. Their frequent use places the highest demands on the safety of this group of drugs. While numerous side effects of antihypertensive drugs have been intensively studied in recent years, the effect of these substances on carcinogenesis is still controversial [3,4,5]. Most importantly, a series of meta-analyses of randomised controlled trials based on aggregate data have investigated the association between class-specific antihypertensive treatment and the risk of cancer, but findings have been conflicting [5]. In a large individual participant data meta-analysis including 260,447 participants with 15,012 cancer events from 33 trials, no associations between any antihypertensive drug class and the risk of any cancer could be identified. However, the authors conclude that available data are still insufficient to entirely rule out an excess risk for cancer, highlighting the need for further studies [5]. We, therefore, used the large electronic medical record database to further analyse the potential association between different antihypertensive therapies and the incidence of cancer diagnoses.

## 2. Methods

### 2.1. Database

This study was based on data from the Disease Analyzer database (IQVIA), which contains drug prescriptions, diagnoses, and basic medical and demographic data obtained directly and in an anonymous format from computer systems used in the practices of general practitioners and specialists [6]. The database covers approximately 3% of all outpatient practices in Germany. Diagnoses (according to the International Classification of Diseases, 10th revision (ICD-10)), prescriptions (according to the Anatomical Therapeutic Chemical (ATC) Classification system), and the quality of reported data are monitored by IQVIA. In Germany, the sampling methods used to select physicians’ practices are appropriate for obtaining a representative database of general and specialized practices [6]. For example, Rathmann et al. could show a good agreement between the incidence or prevalence of cancer diagnoses between the outpatient DA database and German reference data [6]. Finally, this database has already been used in previous studies focusing on antihypertensive therapy [7,8] as well as cancer [9,10].

### 2.2. Study Population

**Figure 1:**
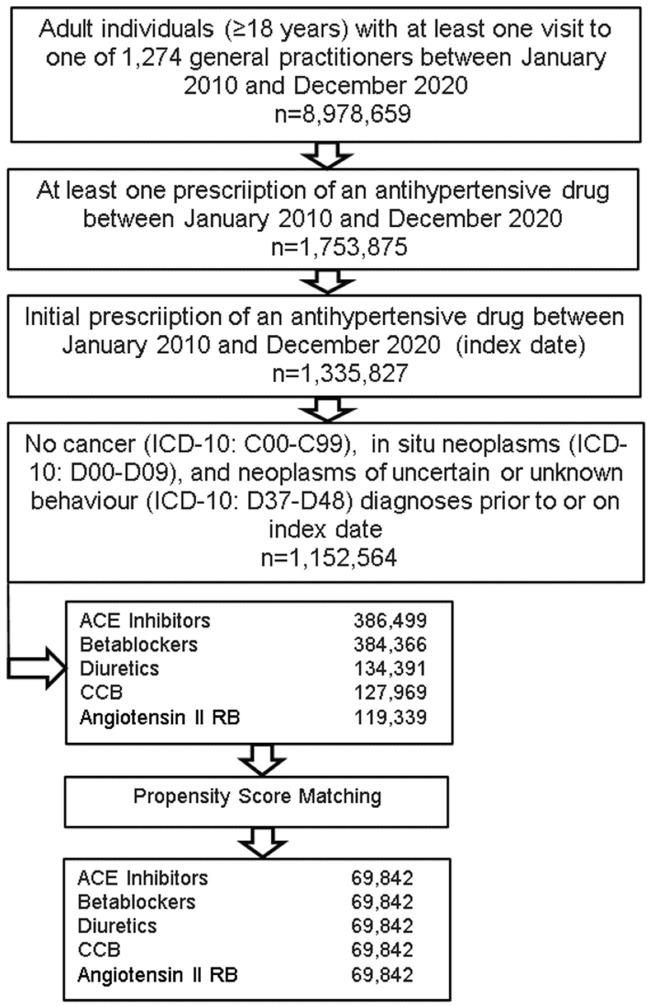
Selection of study patients.

This retrospective cohort study included adult patients (≥18 years) with an initial prescription of antihypertensive therapy (diuretics, ATC: C03A; beta-blockers, ATC: C07A; calcium channel blockers, ATC: C08A; ACE inhibitors, ATC: C09A; angiotensin II receptor blockers, ATC: C09A) in 1,274 general practices in Germany between January 2010 and December 2020 (index date; Figure 1). Patients with previous cancer diagnoses (ICD-10: C00-C99), in situ neoplasms (ICD-10: D00-D09) or neoplasms of uncertain or unknown behavior (ICD-10: D37-D48) prior to or within 90 days from the index date were excluded.

The five antihypertensive drug classes were matched 1:1:1:1:1 to each other patients by propensity scores based on sex, age, index year, and diagnoses documented prior to or on the index date, including obesity (ICD-10: E66), diabetes (ICD-10: E10-E11), lipid metabolism disorder (ICD-10: E78), hypertension (ICD-10: I10), ischemic heart diseases (ICD-10: I20-I25), heart failure (ICD-10: I50), chronic bronchitis or obstructive lung disease (COPD) (ICD-10: J42-J44), and liver diseases (ICD-10: B18, K70-K77).

### 2.3. Study Outcomes and Covariates

The main outcome of the study was the incidence of cancer (ICD 10: C00-C99) in total, as well as cancer of different organs, including digestive organs without liver cancer (ICD 10: C15-C26, excl. C22), liver (ICD-10: C22) respiratory organs (ICD 10: C30-C39), skin (ICD 10: C43, C44), breast (ICD-10: C50), female genital organs (ICD-10: C51-C58), prostate (ICD-10: C60-C63), urinary tract (ICD-10: C64-C68), and lymphoid and haematopoietic tissue (ICD-10: C81-C96) as a function of antihypertensive therapy. Each patient was followed up from day 91 after the index date up to five years until the first cancer diagnosis was documented or the antihypertensive therapy ended (either by switching to another antihypertensive therapy or adding on another drug class to the initial therapy).

### 2.4. Statistical Analyses

Differences in the sample characteristics between the five antihypertensive drug classes were tested using Chi-squared tests for categorical variables and Kruskal–Wallis tests for age. Conditional Cox regression models were conducted to study the association between each antihypertensive drug class as compared to all other antihypertensive drug classes (as a group) and cancer incidence. These models were performed separately for different cancer sites. To counteract the problem of multiple comparisons as well as due to large patient samples, *p*-values < 0.001 were considered statistically significant. Analyses were carried out using SAS version 9.4 (SAS institute, Cary, NC, USA).

## 3. Results

### 3.1. Basic Characteristics of the Study Sample

The present study included 69,842 patients within each therapy group (angiotensin-converting enzyme inhibitors (ACEI), beta-blockers (BB), diuretics (DIU), calcium channel blockers (CCB), and angiotensin-II receptor blockers (ARB); in total: 349,210 patients). The basic characteristics of study patients are displayed in Table 1. Due to the matched-pair study design, all five cohorts had the same age, sex and comorbidity distribution. The mean age (SD) was 65.8 (13.8) years. A total of 56.7% of patients were female. The prevalence of hypertension was 58.1%, while other prevalence rates were 18.4% for diabetes, 12.8% for ischemic heart diseases, and 2.5% for heart failure.

### 3.2. Cumulative Incidence of Cancer Diagnoses

**Figure 2:**
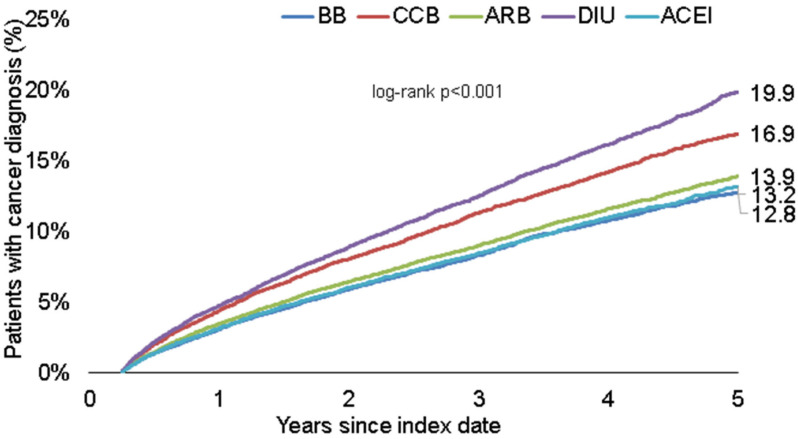
Kaplan–Meier curves showing the time to a cancer diagnosis in patients treated with different antihypertensive drugs. ACEI: ACE inhibitors, BB: beta-blockers, DIU: diuretics, CCB: calcium channel blockers, ARB: angiotensin-II receptor blockers.

We first compared the cumulative incidence of cancer diagnoses between the different treatment groups within five years from the index date. Figure 2 shows the proportion of patients with a documented cancer diagnosis over time. The highest proportion was found in the diuretics group (19.9%), followed by CCB (16.9%). The proportion of patients with a cancer diagnosis was lower among patients receiving treatment with BB (12.8%), ACEI (13.2%) and ARB (13.9%) without reaching significance.

### 3.3. Antihypertensive Therapy and Specific Cancer Site Incidences

We next performed Cox regression analysis to further investigate the association between the different treatment groups and the tumor-site-specific cancer incidence. Here, the use of diuretics was positively correlated with liver cancer incidence (HR: 1.31, 95% CI: 1.12–2.63) and lymphoid/haematopoietic tissue cancer (HR: 1.27, 95% CI: 1.10–1.46, Table 2). Diuretics correlated negatively with prostate (HR: 0.64, 95% CI: 0.53–0.78) and skin cancer incidence (HR: 0.81, 95% CI: 0.72–0.92, Table 2). ARB treatment correlated positively with prostate cancer incidence (HR: 1.50, 95% CI: 1.28–1.65, Table 2). In addition, BB, ACEI and CCB therapy were not significantly associated with an increased or decreased incidence of cancer (Table 2).

## 4. Discussion

In this study based on 349,346 patients, the use of diuretics was positively associated with liver cancer and malignancies of the lymphoid and haematopoietic tissue and negatively associated with prostate cancer and skin cancer. In addition, ARB use was positively associated with prostate cancer (Table 2). Of note, patients receiving CCB had a slightly higher cancer incidence than patients treated with ARB, ACEI or BB (Figure 2), although this value did not reach significance within 5 years of the index date. This finding needs to be further investigated in future studies over a longer observation time, unmasking the specific effects of antihypertensive drugs on different tumor entities.

Propensity score matching ensured that the comorbidities of the patients in each group were comparable. However, a detailed analysis of the underlying diseases, especially liver diseases, was not available, which presents a potential selection bias because patients with liver disease have a different risk of cancer development depending on the type and stage of the liver disease [11]. Furthermore, data on the combined use of multiple antihypertensive drugs were not available, which represents an interfering factor. Another important limitation concerns the study design, which is based on retrospective database analyses. The ICD-10 coding system was used, which sometimes leads to the misclassification and undercoding of certain diagnoses. Finally, data on lifestyle factors, e.g., nicotine use or alcohol intake, were not available in our study.

In women aged 50–75, an increased risk of breast cancer development with diuretic treatment was reported [12]. However, our study did not find an increased risk of breast cancer with diuretics. Other studies point out a possible association of hydrochlorothiazide treatment with an increased risk of non-melanoma skin cancer, whereby study results are heterogeneous [13]. Our findings suggest a decreased incidence of skin cancer with diuretics (Table 2). However, study limitations have been outlined, and a conclusive assessment cannot be made based on our results. Basic studies in rat liver perfusion models have demonstrated an important effect of liver cell hydration on cell proliferation and apoptosis, e.g., hyperosmotic stress upregulates the miR-15/107 family, which downregulates antiapoptotic genes [14]; liver cell swelling has recently been shown to have a substantial effect on hepatocyte proliferation involving miR-141-3p [15]. For thiazide diuretics, ADH-independent water retention has been shown in rats by enhancing water absorption in the inner medullary collecting duct of the kidney [16], potentially contributing to hypoosmolar changes in the serum and leading to a pro-proliferative effect in hepatocytes [15]. However, because of the complex alterations induced by osmotic changes, clinically relevant mechanisms remain to be deciphered in a translational approach, measuring the serum osmolarities in patients treated with different groups of diuretics. As for the mineralocorticoid receptor antagonist spironolactone, hormonal effects might also influence tumor development. In the past, animal studies indicated an increase in myelomonocytic leukaemia with the use of potassium canrenoate, which, as well as spironolactone, is metabolised to canrenone [17]. However, in a large British study, there was no evidence of an increased risk of cancer with spironolactone in humans [17]. Regarding the protective effect of diuretics on prostate cancer development (Table 2), our study confirms findings by other groups, which similarly found a protective effect of certain diuretics on prostate cancer [17,18]. Another meta-analysis of 21 observational studies found no significant relationship between the use of ACEI, ARB, BB and diuretics but of CCB with prostate cancer [19]. Several other studies point towards a protective effect of ARBs on prostate cancer [20,21]. On the contrary, we found a positive association between ARB use and prostate cancer. As it is unclear which patients might be more susceptible to tumor development, these findings will have to be investigated in future prospective trials involving individual patient characteristics and potential genetic profiles. As technical developments are made, pharmacologic treatments are increasingly advancing towards an individualized approach, taking into account individual genetic variants, which potentially influence drug response and the risk of cancer development [22].

Our study has addressed an ongoing controversy about the safety of blood pressure-lowering medication with respect to cancer risk using a large database including patients treated in German outpatient units. While our data suggest increased risks for specific cancers in patients using diuretics, it is important to note the limitations of our study and to understand that our data do not at all justify making or changing therapeutic decisions in patients at this time point. Rather, our study must be seen in the context of numerous, partly contradictory results which, taken together, allow the conclusion that patients using antihypertensive medication according to current national or international guidelines should continue to take their medications to prevent cardiovascular complications of their underlying disease condition. Further data are needed to finally judge whether antihypertensive drugs increase cancer risk.

## Figures and Tables

**Table 1:** Basic characteristics of the study sample after propensity score matching.

Variable	Proportion Affected among Patients Treated with ACEI (%)	Proportion Affected among Patients Treated with BB (%)	Proportion Affected among Patients Treated with DIU (%)	Proportion Affected among Patients Treated with CCB (%)	Proportion Affected among Patients Treated with ARB (%)	*p*-Value
N	69,842	69,842	69,842	69,842	69,842	
Age (Mean, SD)	65.8 (13.8)	65.8 (13.8)	65.8 (13.8)	65.8 (13.8)	65.8 (13.8)	1.000
Age ≤ 60	34.6	34.6	34.6	34.6	34.6	1.000
Age 61–70	24.0	24.0	24.0	24.0	24.0	
Age 71–80	26.9	26.9	26.9	26.9	26.9	
Age > 80	14.5	14.5	14.5	14.5	14.5	
Women	56.7	56.7	56.7	56.7	56.7	1.000
Men	43.3	43.3	43.3	43.3	43.3	
Diabetes	18.4	18.4	18.4	18.4	18.4	1.000
Obesity	11.7	11.7	11.7	11.7	11.7	1.000
Lipid metabolism disorder	20.9	20.9	20.9	20.9	20.9	1.000
Hypertension	58.1	58.1	58.1	58.1	58.1	1.000
Ischemic heart diseases	12.8	12.8	12.8	12.8	12.8	1.000
Heart failure	2.5	2.5	2.5	2.5	2.5	1.000
COPD	6.6	6.6	6.6	6.6	6.6	1.000
Liver diseases	5.1	5.1	5.1	5.1	5.1	1.000

Proportions of patients given in %, unless otherwise indicated. SD: standard deviation.

**Table 2:** Association between antihypertensive therapy and the incidence of cancer (Cox regression models).

Cancers Site	BB vs. Rest	CCB vs. Rest	ARB vs. Rest	DIU vs. Rest	ACEI vs. Rest
Cancer total	0.88 (0.83–0.93)	1.03 (0.98–1.09)	1.10 (1.04–1.16)	0.98 (0.93–1.04)	1.00 (0.95–1.06)
Prostate (men)	0.85 (0.70–1.02)	1.01 (0.85–1.20)	1.50 (1.28–1.65)	0.64 (0.53–0.78)	1.08 (0.91–1.28)
Breast (women)	0.78 (0.62–0.92)	0.93 (0.80–1.08)	1.15 (0.99–1.32)	1.05 (0.91–1.22)	1.10 (0.95–1.26)
Female genital organs (women)	0.70 (0.51–0.97)	0.94 (0.71–1.23)	1.10 (0.84–1.43)	1.36 (1.06–1.74)	0.91 (0.69–1.21)
Skin	0.94 (0.83–1.07)	1.09 (0.98–1.22)	1.14 (1.02–1.27)	0.81 (0.72–0.92)	1.03 (0.92–1.16)
Respiratory organs	0.97 (0.79.1.20)	1.09 (0.90–1.35)	0.91 (0.74–1.12)	1.13 (0.94–1.37)	0.89 (0.73–1.09)
Digestive organs excl. liver	0.80 (0.69–0.93)	1.14 (1.00–1.29)	1.00 (0.87–1.14)	0.97 (0.85–1.11)	1.09 (0.95–1.24)
Liver	1.22 (0.78–1.91)	0.68 (0.40–1.14)	0.79 (0.48–1.30)	1.71 (1.12–2.63)	0.78 (0.48–1.29)
Urinary tract	0.78 (0.53–0.92)	1.22 (1.01–1.48)	1.22 (1.01–1.49)	0.81 (0.66–1.00)	1.05 (0.86–1.29)
Lymphoid and haematopoietic tissue	0.96 (0.82–1.13)	0.88 (0.75–1.03)	1.03 (0.89–1.19)	1.27 (1.10–1.46)	0.88 (0.75–1.02)

ACEI: ACE inhibitors, BB: beta-blockers, DIU: diuretics, CCB: calcium channel blockers, ARB: angiotensin-II receptor blockers.

## Data Availability

The data that support the findings of this study are available on request from the corresponding authors.

## References

[1] Zhou B., Carrillo-Larco R.M., Danaei G., Riley L.M., Paciorek C.J., Stevens G.A., Gregg E.W., Bennett J.E., Solomon B., Singleton R.K. (2021). Worldwide trends in hypertension prevalence and progress in treatment and control from 1990 to 2019: A pooled analysis of 1201 population-representative studies with 104 million participants. Lancet.

[2] Hajjar I., Kotchen J.M., Kotchen T.A. (2006). Hypertension: Trends in prevalence, incidence, and control. Annu. Rev. Public Health.

[3] Battistoni A., Tocci G., Presta V., Volpe M. (2021). Antihypertensive drugs and the risks of cancer: More fakes than facts. Eur. J. Prev. Cardiol..

[4] Singh A., Bangalore S. (2012). Which, if any, antihypertensive agents cause cancer?. Curr. Opin. Cardiol..

[5] Copland E., Canoy D., Nazarzadeh M., Bidel Z., Ramakrishnan R., Woodward M., Chalmers J., Teo K.K., Pepine C.J., Davis B.R. (2021). Antihypertensive treatment and risk of cancer: An individual participant data meta-analysis. Lancet Oncol..

[6] Rathmann W., Bongaerts B., Carius H.J., Kruppert S., Kostev K. (2018). Basic characteristics and representativeness of the German Disease Analyzer database. Int. J. Clin. Pharmacol. Ther..

[7] Jacob L., Kostev K. (2018). Persistence with antihypertensive drugs in patients with depression in Germany. Int. J. Clin. Pharmacol. Ther..

[8] Warda A., Reese J.P., Tanislav C., Kostev K. (2019). The association between antihypertensive therapy and the incidence of Parkinson’s disease in patients followed in general practices in Germany. Int. J. Clin. Pharmacol. Ther..

[9] Loosen S.H., Jördens M.S., Luedde M., Modest D.P., Labuhn S., Luedde T., Kostev K., Roderburg C. (2021). Incidence of Cancer in Patients with Irritable Bowl Syndrome. J. Clin. Med..

[10] Loosen S.H., Roderburg C., Jördens M.S., Fluegen G., Luedde T., Kostev K. (2022). Overweight and Obesity Determine the Risk for Gastrointestinal Cancer in a Sex-Dependent Manner: A Retrospective Cohort Study of 287,357 Outpatients in Germany. Cancers.

[11] Natarajan Y., Kramer J.R., Yu X., Li L., Thrift A.P., El-Serag H.B., Kanwal F. (2020). Risk of Cirrhosis and Hepatocellular Cancer in Patients With NAFLD and Normal Liver Enzymes. Hepatology.

[12] Largent J.A., McEligot A.J., Ziogas A., Reid C., Hess J., Leighton N., Peel D., Anton-Culver H. (2006). Hypertension, diuretics and breast cancer risk. J. Hum. Hypertens..

[13] Garrido P.M., Borges-Costa J. (2020). Hydrochlorothiazide treatment and risk of non-melanoma skin cancer: Review of the literature. Rev. Port. Cardiol..

[14] Santosa D., Castoldi M., Paluschinski M., Sommerfeld A., Häussinger D. (2015). Hyperosmotic stress activates the expression of members of the miR-15/107 family and induces downregulation of anti-apoptotic genes in rat liver. Sci. Rep..

[15] Bardeck N., Paluschinski M., Castoldi M., Kordes C., Görg B., Stindt J., Luedde T., Dahl S.V., Häussinger D., Schöler D. (2022). Swelling-induced upregulation of miR-141-3p inhibits hepatocyte proliferation. JHEP Rep..

[16] César K.R., Magaldi A.J. (1999). Thiazide induces water absorption in the inner medullary collecting duct of normal and Brattleboro rats. Am. J. Physiol..

[17] Mackenzie I.S., Morant S.V., Wei L., Thompson A.M., MacDonald T.M. (2017). Spironolactone use and risk of incident cancers: A retrospective, matched cohort study. Br. J. Clin. Pharmacol..

[18] Hiebert B.M., Janzen B.W., Sanjanwala R.M., Ong A.D., Feldman R.D., Kim J.O. (2021). Impact of spironolactone exposure on prostate cancer incidence amongst men with heart failure: A Pharmacoepidemiological study. Br. J. Clin. Pharmacol..

[19] Cao L., Zhang S., Jia C.M., He W., Wu L.T., Li Y.Q., Wang W., Li Z., Ma J. (2018). Antihypertensive drugs use and the risk of prostate cancer: A meta-analysis of 21 observational studies. BMC Urol..

[20] Uemura H., Ishiguro H., Nagashima Y., Sasaki T., Nakaigawa N., Hasumi H., Kato S., Kubota Y. (2005). Antiproliferative activity of angiotensin II receptor blocker through cross-talk between stromal and epithelial prostate cancer cells. Mol. Cancer Ther..

[21] Rao G.A., Mann J.R., Bottai M., Uemura H., Burch J.B., Bennett C.L., Haddock K.S., Hébert J.R. (2013). Angiotensin receptor blockers and risk of prostate cancer among united states veterans. J. Clin. Pharmacol..

[22] Franceschini N., Chasman D.I., Cooper-DeHoff R.M., Arnett D.K. (2014). Genetics, ancestry, and hypertension: Implications for targeted antihypertensive therapies. Curr. Hypertens. Rep..

